# MTOR inhibition favors the differentiation of human in vitro-induced regulatory T cell through selective protein synthesis

**DOI:** 10.1186/2051-1426-3-S2-P79

**Published:** 2015-11-04

**Authors:** Viviana Volta, Amanda Ernlund, Amanda Valeta, Sandra Demaria, Robert J Schneider

**Affiliations:** 1 NYU School of Medicine, Microbiology Department, New York, NY, USA; 2 NYU School of Medicine, Pathology Department, New York, NY, USA

## 

The immunological composition of the primary cancer, metastatic sites, and stromal tissue determine cancer progression and treatment response. Breast cancers produce cytokines and chemokines that attract and polarize immune cells in a manner that promotes disease progression and metastasis. For example, macrophages are polarized to anti-inflammatory type II tumor activating macrophages (TAMs), and CD4^+^ T cells into tumor promoting, immune suppressing T regulatory cells (Tregs).

Studies in mice and humans show that Tregs also are developed when inhibiting the kinase mTOR (mammalian target of rapamycin) via a poorly understood mechanism. mTOR forms two complexes in the cell, mTORC1 and mTORC2, which regulate multiple metabolic processes. In particular, mTORC1 is inhibited by the immunosuppressant rapamycin and stimulates the translation of mRNAs involved in cell growth and proliferation. Protein synthesis is a highly regulated process involving general mRNA and selective mRNA translation. Our hypothesis is that mTOR downregulation alters lymphocyte gene expression by favoring the translation of specific mRNAs required for Treg differentiation.

We generated human Tregs induced in culture (iTregs) by mTORC1 inhibition through RAD001 (everolimus), a rapalog used as an anti-cancer drug. By contrast, mTORC1/2 dual inhibitor PP242 blocked the growth of all the lymphocytes, pointing to a specific role of mTORC1 in Treg differentiation. In fact, we found that the proliferation of highly suppressive iTregs requires co-treatment with RAD001 and the cytokine TGFβ. Murine models do not require TGFβ, suggesting that caution should be used in extending findings from mouse to human Treg studies. Protein synthesis analysis in double-treated (RAD001+TGFβ), control-treated, RAD001- and TGFβ-treated cells shows that translation is greatly inhibited in the double-treated cells. Genome-wide translation profiling of mRNAs associated with actively translating ribosomes confirmed that only a selective pool of specific mRNAs is translated in the iTreg population. These specific mRNAs may be recruited to ribosome via an mTOR-independent mechanism involving PAIP2, and eIF4G adapter protein, and the ribosomal protein S25.

Our work indicates the importance of selective translational regulation as an additional determinant of gene expression regulating T cell fate. We suggest that iTreg development is facilitated by selective translation of specific mRNAs whose recruitment to the ribosome is augmented when mTORC1 is inhibited. These findings also suggest that cancer treatments causing an improper balance of mTORC1 inhibition might attenuate the anti-tumor immune response through development of Tregs in the tumor microenvironment

